# Myocardial Involvement in Systemic Sclerosis: A State-of-the-Art Review of Multimodality Cardiovascular Imaging

**DOI:** 10.3390/diagnostics16081196

**Published:** 2026-04-17

**Authors:** Mislav Radić, Tina Bečić, Petra Šimac Prižmić, Josipa Radić, Hana Đogaš, Ivona Matulić, Ivana Jukić, Jonatan Vuković, Damir Fabijanić

**Affiliations:** 1 Department of Internal Medicine, Division of Rheumatology, Allergology and Clinical Immunology, University Hospital of Split, 21000 Split, Croatia; petra_simac@hotmail.com; 2 Department of Internal Medicine, School of Medicine, University of Split, 21000 Split, Croatia; jonatan.vukovic@mefst.hr; 3 Department of Cardiovascular Diseases, University Hospital of Split, 21000 Split, Croatia; tina.becic@gmail.com (T.B.); damirfabijanic62@gmail.com (D.F.); 4 Department of Internal Medicine, Division of Nephrology, Dialysis and Arterial Hypertension, University Hospital of Split, 21000 Split, Croatia; 5 Department of Neurology, University Hospital of Split, 21000 Split, Croatia; hana.dogas@gmail.com; 6 Private Clinic Matulic, Osjecka Ulica 24a, 21000 Split, Croatia; ivonamatulic@yahoo.com; 7 Department of Internal Medicine, Division of Gastroenterology, University Hospital of Split, 21000 Split, Croatia; ivjukic@gmail.com; 8 Faculty of Health Sciences, University of Split, 21000 Split, Croatia; 9 Department of Clinical Propedeutics, School of Medicine, University of Split, 21000 Split, Croatia

**Keywords:** systemic sclerosis, myocardial involvement, cardiac involvement, cardiovascular imaging, echocardiography, cardiac magnetic resonance, PET imaging, myocardial fibrosis, microvascular dysfunction, multimodality imaging

## Abstract

Systemic sclerosis (SSc) is a complex autoimmune connective tissue disease characterized by microvascular dysfunction, immune activation, and progressive fibrosis affecting multiple organs, including the heart. Myocardial involvement represents an important but frequently underrecognized manifestation of SSc and may develop even in the absence of overt clinical symptoms. Cardiac manifestations include ventricular dysfunction, arrhythmias, conduction abnormalities, and heart failure, contributing substantially to morbidity and mortality. The underlying pathophysiology involves coronary microvascular dysfunction, immune-mediated myocardial inflammation, and progressive myocardial fibrosis, which often precede clinically apparent cardiac disease. This review aims to summarize the current understanding of myocardial involvement in SSc and to provide a comprehensive overview of contemporary multimodality cardiovascular imaging techniques for its detection, characterization, and risk stratification. A comprehensive overview of the current literature was conducted focusing on established and emerging cardiovascular imaging modalities for the evaluation of myocardial involvement in SSc. Particular attention was given to echocardiography, cardiac magnetic resonance (CMR), nuclear imaging techniques including positron emission tomography (PET) and single-photon emission computed tomography (SPECT), and cardiac computed tomography (CT). Recent advances in imaging biomarkers, parametric mapping, myocardial strain analysis, and emerging technologies such as artificial intelligence (AI), radiomics, and molecular imaging were also considered. Multimodality cardiovascular imaging plays a central role in the early detection and comprehensive assessment of myocardial involvement in SSc. Advanced imaging techniques enable improved identification of subclinical myocardial dysfunction, microvascular impairment, inflammation, and fibrosis. An integrated imaging approach combining echocardiography, CMR, nuclear imaging, and CT may facilitate earlier diagnosis, enhance risk stratification, and ultimately improve cardiovascular outcomes in patients with SSc.

## 1. Introduction: Why Myocardial Involvement in Systemic Sclerosis Is Underdetected

Systemic sclerosis (SSc) is a chronic autoimmune connective tissue disease characterized by immune dysregulation, widespread microvascular dysfunction, and progressive fibrosis affecting multiple organs, including the skin, lungs, gastrointestinal tract, and heart [1,2]. The disease is classified according to the American College of Rheumatology/European League Against Rheumatism (ACR/EULAR) criteria, reflecting its heterogeneous clinical spectrum and multisystem involvement [3]. Cardiac involvement is one of the most serious complications of SSc and is associated with increased morbidity and mortality, although myocardial involvement often remains underrecognized due to its frequently presentation in early stages [4,5]. Structural myocardial abnormalities are more common than clinically overt disease, highlighting a gap between pathological involvement and clinical detection [6,7,8].

A distinctive feature of SSc-related cardiomyopathy is the unique interplay between coronary microvascular dysfunction, diffuse myocardial fibrosis, and arrhythmogenic remodeling. Unlike ischemic cardiomyopathy, myocardial injury in SSc is primarily driven by microvascular abnormalities and immune-mediated processes rather than obstructive epicardial coronary artery disease. This results in diffuse and heterogeneous myocardial involvement, which may predispose to early ventricular dysfunction and increased arrhythmic risk even in the absence of overt structural heart disease.

The pathogenesis of myocardial involvement in SSc is multifactorial and involves coronary microvascular dysfunction, immune-mediated inflammation, and progressive myocardial fibrosis [9,10]. These processes lead to recurrent myocardial injury, ventricular dysfunction, and arrhythmogenic remodeling [5,11]. Histopathological studies have demonstrated patchy myocardial fibrosis and small-vessel vasculopathy as characteristic features of cardiac involvement in SSc [7,12]. Importantly, myocardial dysfunction may be present even in asymptomatic patients, suggesting early subclinical disease [13]. Clinically apparent manifestations, including heart failure and arrhythmias, typically occur later in the disease course [4,14], while cardiovascular complications remain a major contributor to mortality [15,16]. Long-term studies confirm that cardiac and pulmonary vascular involvement are key determinants of survival in SSc [17]. Several factors contribute to the underdetection of myocardial involvement. Conventional diagnostic methods may lack sensitivity for early disease, and symptoms may be nonspecific or masked by other systemic manifestations [4,18]. In addition, myocardial injury often develops through diffuse and heterogeneous processes that are not easily captured by standard techniques [19].

These limitations highlight the need for sensitive imaging techniques capable of detecting early and subclinical myocardial involvement. Advances in cardiovascular imaging have substantially improved the detection of subclinical myocardial involvement in SSc. Techniques such as speckle-tracking echocardiography and cardiac magnetic resonance (CMR) enable identification of early myocardial dysfunction, microvascular impairment, inflammation, and fibrosis [18,19,20]. In particular, CMR techniques, including late gadolinium enhancement (LGE) and parametric mapping, allow for noninvasive assessment of myocardial tissue characteristics [21,22]. Similarly, myocardial strain imaging can detect early ventricular dysfunction even in asymptomatic patients [23,24]. Despite these advances, optimal strategies for screening and monitoring myocardial involvement in SSc remain incompletely defined [25,26]. Given the clinical importance of early detection, there is increasing interest in integrating multimodality cardiovascular imaging into routine clinical evaluation [18,27]. This review therefore aims to provide a focused overview of myocardial involvement in SSc and to highlight the role of contemporary multimodality cardiovascular imaging in its detection, characterization, and clinical management.

A narrative literature search was conducted using PubMed, Scopus, and Web of Science to identify relevant studies published up to 2025. The search strategy included combinations of predefined keywords using Boolean operators, such as (“systemic sclerosis” OR “scleroderma”) AND (“cardiac involvement” OR “myocardial fibrosis” OR “cardiomyopathy”) AND (“echocardiography” OR “cardiac magnetic resonance” OR “CMR” OR “PET” OR “computed tomography”). Studies were screened based on titles and abstracts, followed by full-text evaluation where appropriate. Priority was given to original studies, systematic reviews, and consensus documents focusing on imaging-based assessment of myocardial involvement in systemic sclerosis. Articles were selected based on their relevance to pathophysiology, diagnostic performance, and clinical applicability of imaging modalities. Non-relevant articles, studies not focused on cardiovascular imaging, and publications lacking sufficient methodological clarity were excluded. Additional relevant studies were identified through manual screening of reference lists.

## 2. Pathobiology of Systemic Sclerosis Cardiomyopathy Relevant to Imaging

**Figure 1:**
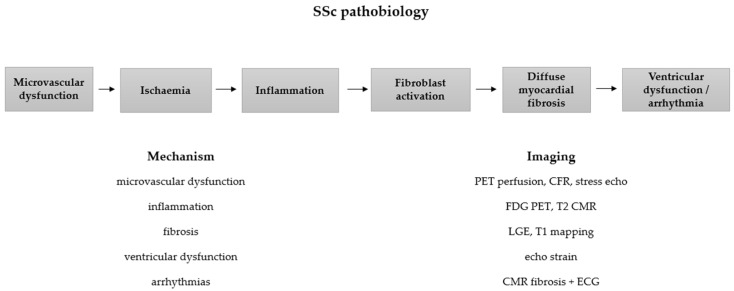
Pathophysiological cascade of SSc cardiomyopathy and corresponding cardiovascular imaging modalities used for detection of myocardial involvement.

Cardiac involvement in SSc results from the interplay of microvascular dysfunction, immune-mediated inflammation, and progressive myocardial fibrosis [1,5]. These processes often develop subclinically and may remain undetected for prolonged periods [13,28]. Understanding these mechanisms is essential because each component can be assessed using specific cardiovascular imaging techniques. Contemporary imaging modalities therefore provide a noninvasive approach to detect and characterize different stages of SSc cardiomyopathy [18,19]. The relationship between key pathogenic mechanisms and corresponding imaging techniques is illustrated in Figure 1.

### 2.1. Microvascular Dysfunction and Ischemia Without Epicardial CAD

Microvascular dysfunction is a central mechanism in SSc cardiomyopathy and is characterized by structural and functional abnormalities of the coronary microcirculation, leading to impaired myocardial perfusion in the absence of obstructive epicardial coronary artery disease [5,9]. These abnormalities result in reduced coronary flow reserve and recurrent ischemic injury, contributing to progressive myocardial fibrosis [7,20]. Noninvasive imaging techniques such as stress echocardiography, myocardial perfusion imaging, and PET enable assessment of coronary microvascular function and detection of perfusion abnormalities [10,29].

### 2.2. Inflammation–Fibrosis Continuum

Immune-mediated myocardial inflammation represents a key component of SSc cardiomyopathy and contributes to myocardial injury through activation of profibrotic pathways [1,11]. This inflammation–fibrosis continuum leads to diffuse or patchy myocardial remodeling, which may impair contractility and promote ventricular dysfunction [5,19]. Cardiac magnetic resonance (CMR), particularly with parametric mapping techniques including T1 and T2 mapping and extracellular volume quantification, enables noninvasive assessment of myocardial inflammation and diffuse fibrosis [21,22].

### 2.3. Conduction System Disease and Arrhythmogenic Substrate

Myocardial fibrosis and microvascular injury contribute to the development of arrhythmogenic substrates in SSc. Structural remodeling disrupts normal electrical conduction pathways and promotes electrical heterogeneity, increasing susceptibility to arrhythmias [12,30]. Imaging techniques, particularly CMR with late gadolinium enhancement and echocardiographic strain imaging, enable identification of structural abnormalities associated with increased arrhythmic risk [20,31].

These pathophysiological mechanisms provide the foundation for the use of advanced cardiovascular imaging techniques aimed at detecting microvascular dysfunction, inflammation, and myocardial fibrosis in SSc.

## 3. Clinical Phenotypes and Outcomes to Be Targeted by Imaging

Cardiac involvement in SSc encompasses a broad spectrum of clinical phenotypes ranging from subclinical myocardial abnormalities to overt heart failure, arrhythmias, and pulmonary hypertension. Because myocardial disease in SSc often evolves gradually and may remain asymptomatic in its early stages, imaging plays a critical role in identifying clinically relevant cardiac phenotypes before irreversible myocardial damage occurs [4,18]. Contemporary cardiovascular imaging therefore aims not only to detect structural myocardial abnormalities but also to identify patients at increased risk of adverse clinical outcomes. Observational cohort studies have shown that cardiac involvement is frequently underestimated in routine clinical evaluation and may only become clinically apparent in advanced stages of the disease [13,28].

One of the most important clinical phenotypes targeted by imaging is subclinical myocardial dysfunction. Numerous studies have demonstrated that patients with SSc may develop early ventricular dysfunction even in the absence of overt cardiac symptoms. Subclinical abnormalities in myocardial mechanics, particularly reduced global longitudinal strain (GLS), may precede detectable reductions in left ventricular ejection fraction and can be identified using speckle-tracking echocardiography [23,24]. These findings are supported by broader echocardiographic research demonstrating that myocardial deformation imaging provides sensitive markers of early myocardial dysfunction before conventional parameters become abnormal [32,33]. Similarly, cardiac magnetic resonance (CMR) studies have shown that diffuse myocardial fibrosis and inflammation may be present even in patients without clinically apparent cardiac disease [19]. Early myocardial abnormalities have also been demonstrated in treatment-naïve patients with connective tissue diseases using CMR, further supporting the concept of subclinical cardiomyopathy [34]. Identification of this early phenotype is important because it may represent a potentially reversible stage of myocardial injury.

Another clinically important phenotype is overt ventricular dysfunction and heart failure. As outlined in Section 2, progressive myocardial fibrosis and microvascular injury represent the key underlying mechanisms contributing to this phenotype. Both left and right ventricular dysfunction have been described in SSc and are associated with adverse clinical outcomes [4,14]. Imaging modalities such as echocardiography and CMR are essential for assessing ventricular function, myocardial structure, and ventricular remodeling, allowing clinicians to differentiate primary myocardial involvement from secondary cardiac complications such as pulmonary hypertension-related right ventricular dysfunction [19,35]. Clinical guidelines emphasize the importance of comprehensive cardiac evaluation in patients with heart failure, including assessment of ventricular function, structural abnormalities, and myocardial tissue characteristics [36,37]. Early recognition of ventricular dysfunction is particularly important because it may influence therapeutic strategies and clinical monitoring.

Pulmonary hypertension-related cardiac involvement represents another major clinical phenotype in SSc. Pulmonary arterial hypertension (PAH) is a well-recognized complication of the disease and contributes significantly to morbidity and mortality [26,38]. Screening strategies have been developed to facilitate early detection of PAH in SSc because of its major impact on survival [39,40]. Imaging plays a central role in screening and risk stratification of patients with suspected PAH, particularly through echocardiographic assessment of right ventricular size, function, and pulmonary pressures [25]. Because right ventricular dysfunction is a major determinant of prognosis in PAH, imaging evaluation of right heart structure and function is essential in the management of these patients. Echocardiographic assessment of right ventricular function and pulmonary hemodynamics has therefore become a key component of screening algorithms for SSc-associated pulmonary vascular disease [41].

Arrhythmias and conduction abnormalities constitute another clinically significant phenotype of SSc cardiac involvement. As previously discussed, myocardial fibrosis and microvascular injury create arrhythmogenic substrates that predispose patients to atrial and ventricular arrhythmias. Fibrotic remodeling of the myocardium can disrupt normal electrical conduction pathways and create heterogeneous conduction properties that increase the risk of ventricular arrhythmias and sudden cardiac death [42]. Imaging modalities such as CMR can detect myocardial fibrosis through LGE, which has been associated with increased arrhythmic risk and adverse cardiovascular outcomes [20,31]. Identifying patients with structural substrates for arrhythmias may therefore facilitate targeted rhythm monitoring and early therapeutic intervention.

Importantly, cardiovascular imaging also plays a key role in predicting long-term clinical outcomes in patients with SSc. Several studies have demonstrated that imaging biomarkers, including impaired myocardial strain, ventricular dysfunction, and myocardial fibrosis, are associated with increased risk of cardiovascular events and mortality [16,23]. Long-term observational studies have also shown that cardiovascular complications remain a major determinant of mortality in SSc [17]. These findings highlight the prognostic value of imaging-based risk stratification in SSc and support the integration of multimodality imaging into routine clinical assessment.

Given the heterogeneity of cardiac manifestations in SSc, a comprehensive imaging strategy is often required to identify the full spectrum of clinically relevant phenotypes. Multimodality imaging approaches integrating echocardiography, CMR, CT, and nuclear imaging may provide complementary information regarding myocardial structure, function, perfusion, inflammation, and coronary anatomy [18,27], as summarized in Table 1.

Advances in cardiovascular imaging have also improved the detection of myocardial abnormalities in rheumatologic diseases, particularly through the use of quantitative CMR techniques and advanced echocardiographic deformation imaging [28,43]. Such integrated assessment is increasingly recognized as an essential component of contemporary evaluation of myocardial involvement in SSc and may improve early detection, prognostic stratification, and clinical management of affected patients.

## 4. Echocardiography for Detection and Monitoring

Echocardiography represents the first-line imaging modality for the assessment of cardiac involvement in SSc because of its wide availability, noninvasive nature, and ability to provide comprehensive information about cardiac structure and function. Conventional and advanced echocardiographic techniques allow for evaluation of ventricular size, systolic and diastolic function, pulmonary pressures, and myocardial mechanics, making echocardiography a key tool for both screening and longitudinal monitoring of cardiac disease in SSc [4,18]. Early echocardiographic studies demonstrated that cardiac abnormalities in SSc may be detected even in patients without overt cardiovascular symptoms, highlighting the value of routine echocardiographic surveillance [13]. Cardiac abnormalities detected by echocardiography in SSc may include ventricular dysfunction, diastolic abnormalities, right ventricular remodeling, and indirect signs of pulmonary hypertension. Importantly, echocardiography can identify early functional changes before the development of overt clinical manifestations, enabling earlier detection of myocardial involvement [24]. Contemporary echocardiographic approaches increasingly incorporate advanced techniques such as speckle-tracking strain imaging and stress echocardiography, which provide additional insights into myocardial mechanics and coronary microvascular function. The growing use of quantitative echocardiographic techniques has further improved the ability to detect subtle myocardial dysfunction in patients with SSc [32,33].

### 4.1. Standard Transthoracic Echocardiography—LV/RV Size and Function, Diastology

Standard transthoracic echocardiography (TTE) is the cornerstone of cardiac evaluation in patients with SSc. It allows for assessment of left and right ventricular size, systolic function, wall motion abnormalities, and valvular structure, as well as estimation of pulmonary pressures. These parameters are essential for identifying both primary myocardial involvement and secondary cardiac complications associated with SSc [4]. Guidelines from professional societies emphasize the importance of standardized echocardiographic measurements when evaluating ventricular structure and function. Parameters such as left ventricular ejection fraction, chamber dimensions, and right ventricular function indices are recommended for routine clinical assessment [35,44]. In addition, recommendations for image acquisition and quantitative evaluation of cardiac mechanics have been developed to improve reproducibility and standardization of echocardiographic measurements [33,45]. In SSc patients, conventional echocardiography may demonstrate subtle ventricular abnormalities, including mild systolic dysfunction, chamber dilation, or regional wall motion abnormalities associated with myocardial fibrosis or microvascular ischemia. Several cohort studies have demonstrated that echocardiographic abnormalities are common in SSc patients even in the absence of overt cardiac symptoms [13]. Right ventricular assessment is particularly important in SSc because right ventricular dysfunction may reflect either primary myocardial involvement or secondary effects of pulmonary hypertension. Echocardiographic parameters such as tricuspid annular plane systolic excursion (TAPSE), right ventricular fractional area change, and right ventricular dimensions are commonly used to evaluate right ventricular function in this population [44]. Advanced echocardiographic techniques such as three-dimensional echocardiography may further improve quantification of right ventricular volumes and function [46]. Although conventional echocardiographic parameters may remain normal in early disease stages, they provide an important baseline for monitoring disease progression and evaluating therapeutic response in patients with established cardiac involvement.

### 4.2. Tissue Doppler Imaging and Diastolic Dysfunction in Systemic Sclerosis

Diastolic dysfunction is one of the most frequently reported echocardiographic abnormalities in patients with SSc and may occur even in the absence of overt systolic dysfunction. Tissue Doppler imaging (TDI) provides a sensitive method for evaluating myocardial relaxation and ventricular filling pressures by measuring myocardial velocities at the mitral annulus [47]. Early studies have demonstrated that patients with SSc often exhibit impaired myocardial relaxation and increased left ventricular filling pressures, reflecting early myocardial involvement related to diffuse fibrosis and microvascular ischemia [4,24]. Tissue Doppler parameters such as reduced early diastolic velocity (e′) and increased E/e′ ratio have been associated with subclinical myocardial dysfunction in this population. Assessment of diastolic function is particularly important because diastolic abnormalities may represent one of the earliest manifestations of SSc cardiomyopathy. Current echocardiographic guidelines recommend a comprehensive evaluation of diastolic function incorporating multiple parameters, including transmitral inflow patterns, tissue Doppler velocities, and left atrial size [47]. Diastolic abnormalities may reflect underlying diffuse myocardial fibrosis and microvascular disease, which are hallmarks of SSc cardiomyopathy [19,48].

### 4.3. Speckle-Tracking Strain Imaging (LV GLS, RV Strain, LA Strain)

Speckle-tracking echocardiography (STE) has emerged as a powerful tool for detecting subtle myocardial dysfunction in SSc. This technique allows for quantitative assessment of myocardial deformation by measuring strain parameters, including GLS, which reflects longitudinal myocardial fiber shortening [49,50]. Standardization of strain imaging methodology has been emphasized by international task force documents to improve inter-vendor reproducibility and clinical applicability [49,50,51,52,53,54,55,56]. Several studies have demonstrated that patients with SSc may exhibit reduced GLS despite preserved left ventricular ejection fraction, suggesting the presence of subclinical myocardial dysfunction [23,24]. Reduced GLS has been associated with myocardial fibrosis detected by cardiac magnetic resonance imaging and may serve as an early marker of SSc cardiomyopathy. Speckle-tracking techniques can also evaluate right ventricular and left atrial strain, providing additional information about cardiac involvement. Right ventricular longitudinal strain has been shown to detect early right ventricular dysfunction and may be particularly useful in patients with SSc-related pulmonary hypertension [52]. Similarly, left atrial strain analysis may provide insights into atrial remodeling and diastolic dysfunction [53,54]. Because of its sensitivity for detecting early myocardial dysfunction, strain imaging is increasingly used for screening and risk stratification in patients with SSc. Quantitative evaluation of myocardial mechanics using speckle-tracking echocardiography has therefore become an important component of modern echocardiographic assessment [32].

### 4.4. Stress Echocardiography, Coronary Flow Reserve, and Microvascular Disease

Stress echocardiography plays an important role in evaluating myocardial ischemia and coronary microvascular dysfunction in patients with SSc. Unlike classical CAD, myocardial ischemia in SSc is often related to microvascular abnormalities rather than epicardial coronary obstruction [5,9]. Stress echocardiography can identify inducible myocardial ischemia and assess CFR, which reflects the capacity of coronary circulation to increase blood flow in response to increased myocardial demand. Reduced CFR has been described in patients with SSc and may indicate microvascular dysfunction affecting the coronary circulation [55]. Assessment of CFR using Doppler echocardiography provides a noninvasive method for evaluating coronary microvascular function. Impaired CFR has been associated with microvascular disease and may contribute to progressive myocardial fibrosis and ventricular dysfunction in SSc [10]. PET-based studies have similarly demonstrated that reduced CFR is associated with adverse cardiovascular outcomes in patients with coronary microvascular dysfunction [29]. Current recommendations support the use of stress echocardiography for evaluating nonischemic heart disease and microvascular dysfunction in appropriate clinical contexts [56].

### 4.5. Screening for Pulmonary Hypertension-TRV, RV Function, Algorithms

Pulmonary hypertension is a major complication of SSc and represents an important cause of morbidity and mortality. Echocardiography plays a central role in screening for pulmonary hypertension by estimating pulmonary artery pressures and evaluating right ventricular structure and function [25,26]. The tricuspid regurgitation velocity (TRV) is a key echocardiographic parameter used to estimate systolic pulmonary artery pressure. Elevated TRV values, particularly when combined with other echocardiographic findings such as right ventricular dilation or dysfunction, may indicate the presence of pulmonary hypertension and prompt further diagnostic evaluation with right heart catheterization [57]. Screening algorithms incorporating echocardiographic parameters and clinical variables have been developed to improve early detection of PAH in SSc. The DETECT algorithm represents one of the most widely used screening strategies and integrates echocardiographic measurements with laboratory and clinical variables to identify patients at risk for pulmonary hypertension [26]. Early screening strategies for pulmonary hypertension have been shown to significantly improve early diagnosis and clinical outcomes in SSc [39,40]. In addition to pulmonary pressure estimation, echocardiography provides valuable information regarding right ventricular adaptation to increased afterload. Assessment of right ventricular size, systolic function, and interventricular septal configuration is essential for evaluating disease severity and predicting clinical outcomes in patients with SSc-associated pulmonary hypertension [25,44]. Given its accessibility and diagnostic versatility, echocardiography remains a cornerstone imaging modality for the detection, risk stratification, and longitudinal monitoring of cardiovascular involvement in SSc.

**Figure 2:**
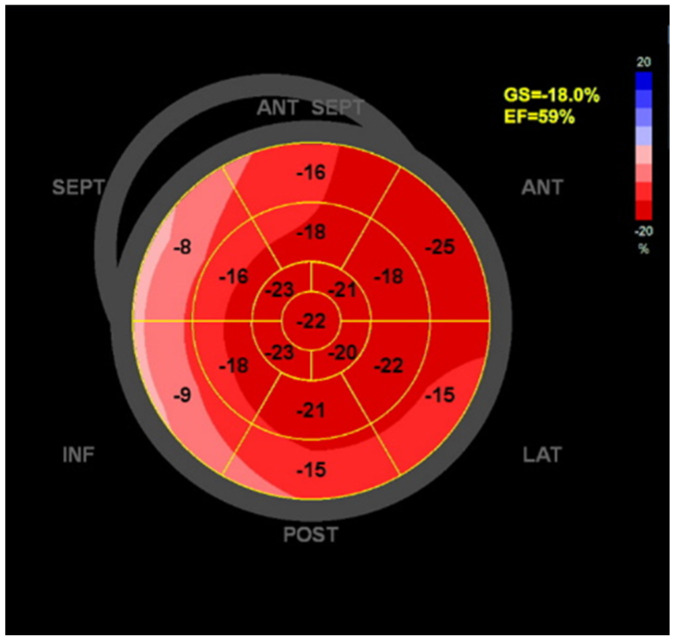
Bull’s-eye plot of left ventricular longitudinal strain demonstrating preserved global strain with regional abnormalities in the septal and inferior segments, reflecting early subclinical and heterogeneous myocardial involvement in systemic sclerosis. Negative values are expressed using standard strain convention.

For example, patients with SSc may present with preserved left ventricular ejection fraction and preserved global longitudinal strain, but with regional abnormalities detected by speckle-tracking echocardiography, particularly in the septal and inferior segments. This pattern reflects early subclinical myocardial involvement and the heterogeneous distribution of myocardial injury characteristic of systemic sclerosis. Such findings may precede overt structural abnormalities and are associated with an increased risk of disease progression (Figure 2).

## 5. Cardiovascular Magnetic Resonance (CMR): Tissue Characterization

CMR has emerged as the reference non-invasive imaging modality for the comprehensive assessment of myocardial involvement in SSc as emphasized in recent comprehensive reviews of cardiac involvement in SSc [58]. Importantly, it plays a central role in the detection of both focal and diffuse myocardial fibrosis, which represents a key feature of SSc cardiomyopathy. In contrast to echocardiography, which primarily evaluates cardiac function, CMR provides multiparametric tissue characterization enabling detection of myocardial inflammation, edema, ischemia, and fibrosis, even in asymptomatic patients [18,28]. Because myocardial involvement in SSc is often patchy and subclinical, CMR is particularly valuable for identifying early disease stages before overt ventricular dysfunction develops [19,59]. The expanding role of CMR in rheumatologic diseases has been emphasized in several consensus statements highlighting its ability to identify early myocardial abnormalities in inflammatory and fibrotic cardiomyopathies [28,43]. Standard CMR protocols include cine imaging for ventricular volumes and function, LGE imaging for focal fibrosis, and parametric mapping techniques such as native T1, T2, and ECV mapping for the assessment of diffuse myocardial abnormalities [21,43]. These techniques are increasingly standardized through international consensus recommendations to improve reproducibility and diagnostic accuracy [43,60]. This multiparametric approach enables characterization of the inflammation–fibrosis continuum that underlies SSc cardiomyopathy.

### 5.1. Ventricular Function and Morphology

CMR cine imaging provides highly reproducible measurements of left and right ventricular volumes, mass, and systolic function and is considered the gold standard for quantitative assessment of cardiac morphology [43,60]. In patients with SSc, CMR may reveal subtle ventricular remodeling even when conventional echocardiographic parameters appear normal. Left ventricular systolic dysfunction is relatively uncommon in early SSc but may develop with progressive myocardial fibrosis and microvascular ischemia [2,5]. More frequently, CMR demonstrates mild reductions in ventricular function or abnormalities in ventricular geometry reflecting diffuse myocardial involvement [34]. Right ventricular dysfunction may also be present and can result from either primary myocardial disease or secondary effects of pulmonary hypertension [52]. Importantly, CMR allows for detection of myocardial involvement before overt heart failure develops. Subclinical abnormalities in ventricular function have been reported even in asymptomatic SSc patients, supporting the role of CMR in early disease detection and risk stratification [19,59]. Several studies have also demonstrated that myocardial abnormalities detected by CMR may already be present at the time of diagnosis of connective tissue diseases, emphasizing the value of early imaging evaluation [34].

### 5.2. LGE Patterns and Prognostic Meaning

LGE imaging enables the identification of focal myocardial fibrosis by exploiting differences in contrast washout between normal and diseased myocardium [61]. In SSc, LGE typically demonstrates non-ischemic patterns, most commonly located in the mid-myocardial or subepicardial layers, reflecting replacement fibrosis related to microvascular injury rather than epicardial CAD. Early studies using LGE-CMR demonstrated that myocardial fibrosis is present in a substantial proportion of SSc patients, even in the absence of clinical cardiac symptoms [20]. The distribution of fibrosis is frequently patchy and may involve the basal and mid-ventricular segments, consistent with the heterogeneous microvascular pathology characteristic of the disease [34]. Other imaging studies have confirmed that focal myocardial fibrosis detected by CMR may correspond to areas of microvascular ischemic injury and fibrotic remodeling [48]. The presence and extent of LGE have important prognostic implications. In various non-ischemic cardiomyopathies, myocardial fibrosis detected by LGE has been associated with increased risk of ventricular arrhythmias, heart failure progression, and mortality [31,62]. Similar observations are emerging in connective tissue diseases, where distinct cardiovascular phenotypes identified by CMR have been shown to be associated with clinical outcomes in systemic sclerosis [63]. Recent systematic evidence further supports the prognostic value of CMR-derived parameters in systemic sclerosis [64], where myocardial fibrosis may serve as a marker of adverse cardiac outcomes [28,59], consistent with broader CMR-based prognostic frameworks established in non-ischemic cardiomyopathies [65]. Therefore, LGE imaging represents a key tool for risk stratification in SSc-related cardiomyopathy. In addition, although epicardial coronary artery disease is not the predominant mechanism of myocardial injury in SSc, CMR may also help identify ischemic myocardial damage and scar in selected patients. This may be particularly relevant in individuals with cardiovascular risk factors or atypical clinical presentations, where differentiation between microvascular disease and concomitant ischemic heart disease is clinically important [66].

### 5.3. T1 Mapping and Extracellular Volume—Diffuse Fibrosis

Although LGE is effective for detecting focal fibrosis, it is relatively insensitive to diffuse myocardial involvement. Parametric mapping techniques, particularly native T1 mapping and ECV quantification, allow for detection of diffuse interstitial fibrosis that may be present even when LGE findings are absent [21,67]. Native T1 mapping measures intrinsic myocardial relaxation properties and increases in the presence of fibrosis, edema, or inflammation [68]. Advances in myocardial mapping techniques, including shortened modified Look-Locker inversion recovery (ShMOLLI), have significantly improved the feasibility and clinical applicability of myocardial T1 mapping [69]. ECV mapping provides a quantitative estimate of the extracellular matrix expansion and is considered a robust marker of diffuse myocardial fibrosis [70,71]. In SSc, several studies have demonstrated elevated native T1 and ECV values compared with healthy controls, indicating diffuse myocardial fibrosis even in patients without overt cardiac disease [19]. These abnormalities correlate with disease severity and may precede structural cardiac remodeling [72]. Increased ECV has also been associated with markers of disease activity and skin fibrosis, suggesting shared pathogenic mechanisms involving fibroblast activation and extracellular matrix deposition [72,73]. Because diffuse myocardial fibrosis represents a key mechanism of SSc cardiomyopathy, T1 mapping and ECV quantification are increasingly recognized as promising imaging biomarkers for early disease detection and risk stratification [59,73]. Quantitative CMR biomarkers may also serve as potential surrogate endpoints in clinical trials evaluating antifibrotic therapies in SSc [28]. Importantly, these CMR-derived parameters are not merely technical measurements but represent clinically relevant biomarkers of myocardial involvement in SSc. LGE enables detection of focal replacement fibrosis associated with adverse outcomes, while native T1 and ECV provide quantitative assessment of diffuse interstitial fibrosis. Together, these techniques allow for early diagnosis, improved risk stratification, and longitudinal monitoring of disease progression in patients with SSc.

### 5.4. T2 Mapping, Myocardial Edema, and Myocarditis Criteria

Inflammatory myocardial injury may occur in SSc as part of the early inflammatory phase preceding fibrosis. CMR techniques sensitive to tissue water content, particularly T2 mapping, enable detection of myocardial edema, which is a hallmark of active inflammation [21]. Elevated T2 values suggest the presence of myocardial edema and may indicate active myocarditis or inflammatory cardiomyopathy. Inflammatory myocardial involvement has been documented in connective tissue diseases using multiparametric CMR, supporting the concept of an inflammation–fibrosis continuum in SSc cardiomyopathy [34]. The diagnostic framework for CMR assessment of myocarditis is based on the Lake Louise Criteria, originally described using T2-weighted imaging and LGE [74] and subsequently updated to incorporate quantitative mapping techniques such as T1 and T2 mapping [22]. According to the updated criteria, the presence of at least one T2-based marker of edema and one T1-based marker of myocardial injury (including LGE or elevated native T1/ECV) supports the diagnosis of myocardial inflammation. These advanced mapping techniques provide improved sensitivity for detecting inflammatory myocardial disease and may identify potentially reversible stages of myocardial involvement before irreversible fibrosis develops [22,75]. Importantly, standardized imaging protocols and mapping recommendations have been developed to facilitate the clinical application of CMR in inflammatory cardiomyopathies [21,43]. Consequently, CMR plays a central role not only in diagnosis but also in monitoring disease activity and therapeutic response in patients with SSc-related cardiac involvement.

**Figure 3:**
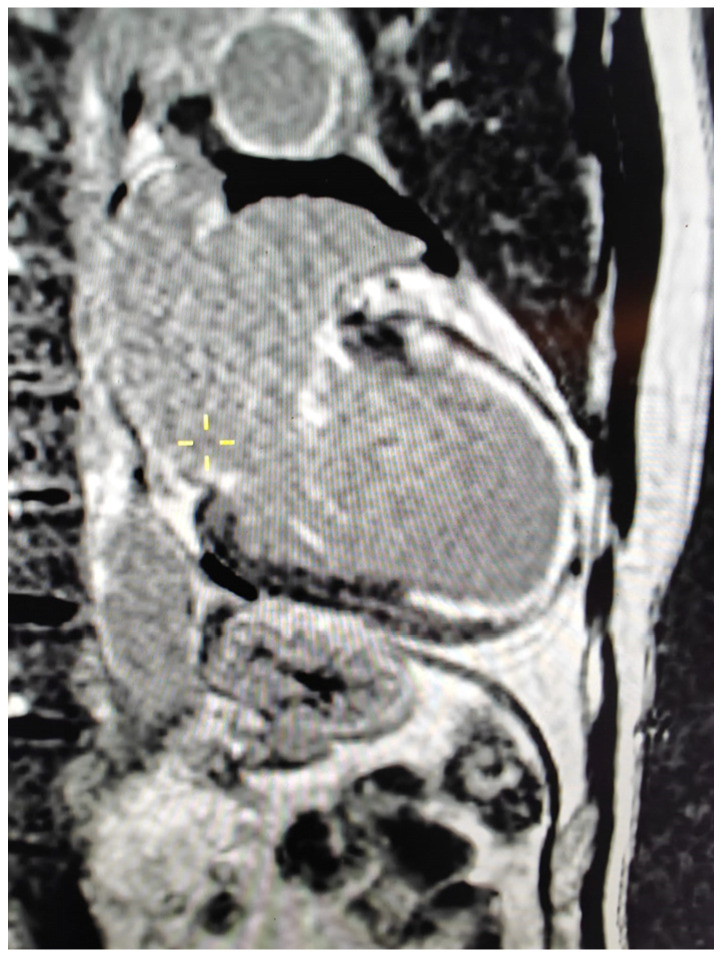
Late gadolinium enhancement (CMR) demonstrating focal non-ischemic myocardial fibrosis with a mid-myocardial/subepicardial pattern, reflecting heterogeneous myocardial involvement characteristic of systemic sclerosis.

For instance, CMR may reveal patchy mid-myocardial or subepicardial late gadolinium enhancement in the absence of obstructive coronary artery disease, consistent with non-ischemic myocardial fibrosis. In addition, elevated native T1 values and increased extracellular volume may indicate diffuse interstitial fibrosis even in asymptomatic patients (Figure 3).

## 6. Cardiac CT: Coronary Assessment and Structural Adjuncts

Cardiac computed tomography (CT) has become an important complementary imaging modality in the evaluation of patients with SSc, particularly for the assessment of coronary artery anatomy and structural cardiac abnormalities. Although myocardial involvement in SSc is primarily related to microvascular dysfunction and diffuse myocardial fibrosis rather than obstructive epicardial CAD, exclusion of significant coronary stenosis is often necessary when patients present with chest pain, ventricular dysfunction, or imaging findings suggestive of ischemia [5,9]. In this context, coronary CT angiography (CCTA) provides a noninvasive method for evaluating epicardial coronary arteries with high diagnostic accuracy. Advances in multidetector CT technology have significantly improved spatial and temporal resolution, allowing for reliable visualization of coronary arteries and plaque morphology [76,77]. CCTA enables detailed visualization of coronary anatomy and allows for reliable exclusion of obstructive CAD in patients with low to intermediate pre-test probability of ischemic heart disease. Current guidelines recognize coronary CT as an appropriate first-line imaging modality for anatomical evaluation of suspected chronic coronary syndromes because of its high negative predictive value for ruling out significant coronary stenosis [78]. Large multicenter trials have demonstrated that CCTA improves diagnostic accuracy and clinical decision-making in patients with suspected CAD [79,80]. In patients with SSc, this capability is particularly relevant because symptoms of myocardial ischemia may reflect either microvascular disease or concomitant atherosclerotic CAD, especially in older patients or those with cardiovascular risk factors [9]. Beyond coronary artery assessment, cardiac CT can provide additional structural information that may be useful in the evaluation of SSc-related cardiac involvement. CT imaging allows for accurate assessment of cardiac chamber size, pericardial anatomy, and extracardiac thoracic structures, including pulmonary parenchyma and pulmonary vasculature. These features are particularly relevant in SSc, where pulmonary fibrosis and pulmonary hypertension frequently coexist with cardiac disease and may contribute to right ventricular remodeling and functional impairment [1,25]. High-resolution CT of the chest is widely used to evaluate interstitial lung disease in SSc and may provide complementary information regarding cardiopulmonary interactions [81,82]. Cardiac CT may also be used to identify structural abnormalities of the pericardium and great vessels. Pericardial thickening or effusion may occur in SSc and can be detected with high spatial resolution using CT imaging [18]. Furthermore, CT-based assessment of coronary artery calcium provides a quantitative measure of coronary atherosclerotic burden and may help refine cardiovascular risk assessment [83,84]. In patients with autoimmune diseases, coronary calcium scoring may reveal accelerated atherosclerosis related to chronic systemic inflammation [85]. More advanced CT techniques, including CT myocardial perfusion imaging, have been developed to evaluate myocardial perfusion and detect ischemia. Dynamic CT perfusion imaging allows for assessment of myocardial blood flow and may help identify perfusion abnormalities associated with coronary microvascular dysfunction [86,87]. Although these approaches are increasingly used in patients with suspected CAD, their role in SSc remains less well established. Nevertheless, CT perfusion imaging has the potential to identify perfusion abnormalities related to coronary microvascular dysfunction, which represents a key mechanism of myocardial injury in SSc [10]. Despite these advantages, cardiac CT has several limitations in the assessment of myocardial involvement in SSc. In particular, CT lacks the capability for detailed myocardial tissue characterization that is provided by CMR imaging, including detection of diffuse fibrosis and myocardial inflammation [18,28]. Additionally, exposure to ionizing radiation and the need for iodinated contrast agents may limit its routine use in some patients. Ongoing technological developments, including photon-counting CT and improved reconstruction algorithms, may help reduce radiation exposure while improving image quality [88]. Overall, cardiac CT serves primarily as an adjunct imaging modality in the evaluation of SSc-related cardiac disease. Its principal role lies in the noninvasive exclusion of obstructive epicardial CAD and in the assessment of structural thoracic abnormalities that may influence cardiac function. When integrated with other imaging modalities such as echocardiography, CMR, and nuclear imaging, cardiac CT contributes to a comprehensive multimodality approach to the evaluation of myocardial involvement in SSc [18,27].

## 7. Nuclear Imaging: PET/SPECT for Inflammation, Perfusion and Fibrosis Activity

Nuclear imaging techniques, including PET and SPECT, provide unique insights into myocardial perfusion, inflammation, and metabolic activity. In SSc, where myocardial injury often results from microvascular dysfunction, immune-mediated inflammation, and progressive fibrosis, nuclear imaging offers complementary information to echocardiography and CMR by enabling functional and molecular assessment of myocardial disease [18,27]. Nuclear imaging also allows for visualization of cellular and molecular processes underlying cardiovascular disease, including inflammatory cell infiltration and fibroblast activation [89]. Unlike anatomical imaging modalities, nuclear techniques allow for evaluation of biological processes underlying myocardial injury, including inflammatory activity, perfusion abnormalities, and fibroblast activation. These features are particularly relevant in SSc cardiomyopathy, which is characterized by a complex interplay between microvascular ischemia, inflammation, and fibrotic remodeling [5,9]. Consequently, PET and SPECT imaging have emerged as promising tools for identifying subclinical myocardial involvement and improving risk stratification in patients with SSc.

### 7.1. 18F-FDG PET for Myocardial Inflammation

Fluorine-18 fluorodeoxyglucose positron emission tomography (18F-FDG PET) is a well-established imaging modality for detecting myocardial inflammation. FDG uptake reflects increased glucose metabolism in activated inflammatory cells, enabling visualization of inflammatory myocardial processes [90]. In inflammatory cardiomyopathies, including myocarditis and autoimmune-mediated myocardial injury, increased FDG uptake corresponds to areas of inflammatory cell infiltration and metabolic activity [91]. Hybrid PET imaging techniques combining metabolic and anatomical information have further improved the diagnostic accuracy of PET in inflammatory cardiac diseases [92].

In SSc, FDG-PET has been explored as a potential tool for detecting early inflammatory myocardial involvement. Pilot studies have demonstrated that patients with SSc may exhibit focal or diffuse myocardial FDG uptake even in the absence of overt cardiac symptoms, suggesting the presence of subclinical inflammatory myocardial injury [93]. These findings support the concept that myocardial inflammation may represent an early stage in the disease process preceding irreversible fibrosis. Accurate interpretation of FDG-PET imaging requires careful patient preparation to suppress physiological myocardial glucose uptake and improve detection of pathological inflammatory activity [94]. Standardized imaging protocols and dietary preparation strategies have been developed to improve suppression of physiological myocardial glucose metabolism and enhance diagnostic accuracy [95]. When appropriately performed, FDG-PET may provide valuable information regarding active myocardial inflammation and may be useful for monitoring inflammatory cardiomyopathies or evaluating treatment response in selected cases [90]. Despite its potential, the role of FDG-PET in SSc remains largely investigational, and further studies are required to establish its clinical utility for routine screening or monitoring of myocardial inflammation in this population.

### 7.2. Perfusion SPECT/PET and Microvascular Ischemia

Myocardial perfusion imaging using SPECT or PET provides important information regarding coronary circulation and myocardial blood flow. In SSc, myocardial ischemia often results from coronary microvascular dysfunction rather than obstructive epicardial CAD [9,10]. Consequently, perfusion imaging techniques may help identify microvascular ischemia that may not be detectable using conventional coronary angiography. SPECT myocardial perfusion imaging has traditionally been used to evaluate regional perfusion abnormalities and detect inducible ischemia. Perfusion defects observed on SPECT imaging in SSc patients may reflect impaired coronary microvascular function and repeated episodes of myocardial ischemia contributing to progressive myocardial fibrosis [5]. However, SPECT provides only relative perfusion assessment and may be limited in detecting diffuse microvascular abnormalities. Advances in SPECT technology, including cadmium-zinc-telluride detectors, have improved sensitivity and reduced radiation exposure [96]. PET myocardial perfusion imaging offers several advantages over SPECT, including higher spatial resolution and the ability to quantify absolute myocardial blood flow and CFR. Quantitative PET imaging allows for detection of global reductions in myocardial perfusion that may indicate coronary microvascular dysfunction [29,97]. PET tracers such as 13N-ammonia, 82Rb, and 15O-water enable highly accurate quantification of myocardial blood flow and coronary physiology [98]. Reduced CFR has been associated with adverse cardiovascular outcomes and may represent an early marker of microvascular disease [29]. Quantitative PET assessment of myocardial blood flow has therefore emerged as an important tool for evaluating coronary microvascular dysfunction and predicting cardiovascular risk [99]. Recent procedural guidelines emphasize the growing role of PET myocardial perfusion imaging in the assessment of coronary physiology and microvascular disease [100,101]. In patients with SSc, PET-based quantification of myocardial blood flow may therefore provide valuable insights into the microvascular component of SSc cardiomyopathy.

### 7.3. FAPI PET as Marker of Fibroblast Activation and Fibrosis Activity

Fibroblast activation protein (FAP)–targeted PET imaging represents an emerging molecular imaging technique for assessing fibroblast activation and fibrotic activity within the myocardium. Radiotracers targeting FAP, such as 68Ga-FAPI, bind to activated fibroblasts involved in tissue remodeling and fibrosis [102,103]. These tracers were initially developed for oncologic imaging but have recently been applied to cardiovascular diseases characterized by fibrotic remodeling [104]. In cardiovascular disease, activated cardiac fibroblasts play a central role in extracellular matrix deposition and myocardial fibrosis. FAPI-PET imaging therefore provides a unique opportunity to visualize active fibrotic remodeling rather than established scar tissue [105]. Experimental and clinical studies have demonstrated that FAPI uptake correlates with areas of active fibroblast proliferation and extracellular matrix production [106]. This distinction is particularly important in diseases such as SSc, where ongoing fibroblast activation contributes to progressive myocardial fibrosis. Molecular imaging approaches capable of identifying active fibrotic remodeling may therefore help distinguish early reversible stages of disease from irreversible myocardial scarring. Although clinical experience remains limited, early studies suggest that FAPI-PET may allow for detection of fibroblast activation in various cardiovascular conditions, potentially identifying stages of active fibrotic remodeling before irreversible myocardial fibrosis develops [105]. In the context of SSc, FAPI-PET may therefore represent a promising future tool for evaluating disease activity and monitoring antifibrotic therapies. At present, however, FAPI-based cardiac imaging remains primarily investigational, and further research is required to determine its diagnostic and prognostic value in patients with SSc.

## 8. Rhythm Monitoring and Imaging: Arrhythmias and Sudden Cardiac Death Risk

Cardiac rhythm disturbances represent a major clinical manifestation of myocardial involvement in SSc and contribute significantly to morbidity and mortality in this patient population. Arrhythmias in SSc arise from a combination of structural myocardial abnormalities, microvascular ischemia, and fibrosis affecting both the myocardium and the cardiac conduction system [2,4]. These pathological processes create electrophysiological substrates that predispose patients to atrial and ventricular arrhythmias, conduction abnormalities, and, in severe cases, sudden cardiac death. Histopathological studies have demonstrated that myocardial fibrosis and small-vessel vasculopathy are common findings in SSc and frequently involve regions of the conduction system [7,12]. Progressive fibrotic remodeling may disrupt normal electrical conduction pathways and produce areas of conduction delay or block, thereby facilitating the development of reentrant arrhythmias. Clinically, patients with SSc may develop a broad spectrum of rhythm disturbances, including atrial fibrillation, supraventricular tachyarrhythmias, atrioventricular conduction block, and ventricular tachyarrhythmias [4]. Electrocardiographic abnormalities are commonly observed in patients with SSc and may include bundle branch block, atrioventricular conduction delay, and ventricular ectopy. These abnormalities often reflect underlying structural myocardial disease and may precede overt clinical manifestations of cardiac involvement [2]. Continuous rhythm monitoring using ambulatory electrocardiography or implantable loop recorders can help identify clinically significant arrhythmias that may otherwise remain undetected during routine clinical evaluation. Advanced cardiovascular imaging plays an increasingly important role in identifying structural substrates associated with arrhythmogenic risk. In particular CMR with LGE enables visualization of focal myocardial fibrosis, which has been associated with ventricular arrhythmias and adverse cardiac outcomes in several cardiomyopathies [31]. In SSc, LGE-detected myocardial fibrosis may reflect areas of scar formation that serve as potential arrhythmogenic foci [20]. Similarly, diffuse myocardial fibrosis detected using parametric mapping techniques may contribute to electrical heterogeneity and increased susceptibility to arrhythmias. Imaging findings may therefore complement rhythm monitoring strategies in identifying patients at increased risk of malignant arrhythmias or sudden cardiac death. Multimodality imaging approaches integrating echocardiography, CMR, and nuclear imaging can help characterize structural myocardial abnormalities, ventricular dysfunction, and inflammatory activity that may contribute to arrhythmogenesis [18,27]. Identification of these imaging biomarkers may facilitate earlier recognition of high-risk patients and guide more intensive rhythm surveillance. Current clinical guidelines emphasize the importance of evaluating structural heart disease when assessing arrhythmic risk. In patients with ventricular arrhythmias or cardiomyopathy, identification of myocardial fibrosis and ventricular dysfunction has important prognostic implications and may influence decisions regarding implantable cardioverter-defibrillator (ICD) therapy [30,107]. Although specific risk stratification strategies for sudden cardiac death in SSc remain incompletely defined, the integration of rhythm monitoring and advanced imaging may improve early detection of arrhythmogenic substrates. Overall, the combined use of rhythm monitoring and multimodality imaging represents an important strategy for evaluating arrhythmias and sudden cardiac death risk in patients with SSc. Improved identification of arrhythmogenic myocardial substrates may enable earlier intervention and contribute to better clinical outcomes in this high-risk population.

## 9. Integrated Multimodality Algorithms

From a clinical perspective, different imaging modalities in SSc should be considered complementary rather than interchangeable, with each technique providing specific information at different stages of disease. Echocardiography, particularly with strain imaging, is most suitable for initial screening and early detection of subclinical myocardial dysfunction. CMR plays a central role in confirming myocardial involvement and in detailed tissue characterization, enabling identification of myocardial fibrosis and inflammation and supporting risk stratification. Nuclear imaging techniques, including PET, provide additional value in selected patients by assessing coronary microvascular dysfunction and inflammatory activity, particularly when findings from other modalities are inconclusive. Cardiac CT is primarily used to exclude obstructive coronary artery disease in patients with suspected ischemia or relevant cardiovascular risk factors. This integrated approach allows for more precise selection of imaging modalities based on clinical presentation and stage of disease. This approach provides a clinically oriented framework for selecting the most appropriate imaging modality in patients with SSc.

Given the heterogeneous mechanisms underlying myocardial involvement in SSc, no single imaging modality can fully characterize the spectrum of cardiac abnormalities associated with the disease. Instead, an integrated multimodality imaging approach combining echocardiography, CMR, CT, and nuclear imaging has increasingly been proposed for comprehensive evaluation of SSc cardiomyopathy [18,27]. Each imaging technique provides complementary information regarding myocardial structure, function, perfusion, inflammation, and fibrosis, allowing clinicians to better identify subclinical disease, monitor disease progression, and stratify cardiovascular risk. Multimodality imaging algorithms are particularly relevant in SSc because myocardial involvement often develops insidiously and may remain clinically silent until advanced myocardial damage has occurred [4]. Early identification of myocardial abnormalities through coordinated imaging strategies may therefore improve patient outcomes by enabling earlier therapeutic intervention and closer monitoring of disease progression.

### 9.1. Detection—Subclinical Disease

Early detection of subclinical myocardial involvement is a key objective of cardiovascular imaging in SSc. Many patients with SSc develop myocardial abnormalities before the onset of overt cardiac symptoms, highlighting the importance of sensitive screening strategies [18,19]. TTE is generally considered the first-line imaging modality for cardiac screening in patients with SSc because of its accessibility and ability to assess ventricular function, diastolic parameters, and pulmonary pressures [4,35]. Advanced echocardiographic techniques such as speckle-tracking strain imaging can detect subtle impairments in myocardial mechanics, including reduced GLS, which may represent an early marker of myocardial dysfunction [23,24]. When echocardiographic findings raise suspicion for myocardial involvement or when clinical risk factors are present, CMR provides a more detailed assessment of myocardial structure and tissue characteristics. CMR techniques including LGE and parametric mapping enable detection of focal and diffuse myocardial fibrosis as well as myocardial inflammation [19,21]. Nuclear imaging techniques, particularly PET-based myocardial perfusion imaging, may further identify coronary microvascular dysfunction that contributes to myocardial injury in SSc [10,29]. The integration of these imaging modalities may therefore facilitate earlier recognition of subclinical myocardial disease and improve screening strategies for patients with SSc.

### 9.2. Monitoring—Therapy Response and Disease Progression

In addition to early detection, cardiovascular imaging plays an important role in monitoring disease progression and assessing therapeutic response in patients with SSc-related cardiac involvement. Because myocardial injury in SSc often evolves gradually through inflammatory and fibrotic processes, serial imaging evaluation can provide valuable insights into disease dynamics [18]. Echocardiography remains the primary modality for longitudinal follow-up because it allows for repeated assessment of ventricular function, diastolic parameters, and pulmonary pressures without radiation exposure. Changes in parameters such as ventricular function, myocardial strain, and right ventricular performance may indicate progression of myocardial disease or the development of complications such as pulmonary hypertension [23,25]. CMR offers additional value for monitoring myocardial tissue characteristics over time. Quantitative techniques such as native T1 mapping and ECV measurement allow for noninvasive evaluation of diffuse myocardial fibrosis and may provide sensitive markers of disease progression or response to antifibrotic or immunomodulatory therapies [72,73]. Similarly, T2 mapping and PET imaging may help assess inflammatory activity and identify patients who could benefit from targeted anti-inflammatory treatment [22,90]. Therefore, multimodality imaging strategies may provide a comprehensive framework for longitudinal monitoring of cardiac involvement in SSc.

### 9.3. Prediction—Risk Stratification and Clinical Outcomes

Beyond diagnosis and monitoring, imaging biomarkers derived from multimodality imaging play an increasingly important role in predicting clinical outcomes in SSc. Several imaging parameters have been associated with adverse cardiovascular events, including myocardial fibrosis detected by CMR, impaired myocardial strain, ventricular dysfunction, and abnormalities in myocardial perfusion [16,18] with key imaging biomarkers and their prognostic implications summarized in Table 2.

In particular, the presence and extent of myocardial fibrosis detected by LGE on CMR have been linked to increased risk of ventricular arrhythmias, heart failure, and mortality in various cardiomyopathies [31]. Similar associations are emerging in SSc, where myocardial fibrosis may represent an important prognostic biomarker of disease severity and arrhythmic risk [20,59]. Myocardial strain abnormalities identified by speckle-tracking echocardiography have also demonstrated prognostic value. Reduced GLS has been associated with increased risk of cardiovascular events and mortality in patients with SSc, even when conventional measures of ventricular function remain preserved, including evidence from CMR feature-tracking studies [23,108], consistent with broader observations demonstrating that GLS provides incremental prognostic value beyond left ventricular ejection fraction in cardiomyopathies [109]. Furthermore, PET-based quantification of myocardial blood flow and CFR may provide prognostic information regarding coronary microvascular dysfunction, which has been linked to adverse cardiovascular outcomes [29,97]. These findings highlight the potential value of integrating functional and molecular imaging biomarkers into risk stratification strategies. Overall, the integration of multimodality imaging findings with clinical assessment and rhythm monitoring may enable more accurate risk stratification in patients with SSc. Such an approach may facilitate earlier identification of high-risk individuals and guide personalized management strategies aimed at preventing cardiovascular complications and improving long-term outcomes. To further improve clinical applicability, the relative positioning of imaging modalities across different clinical scenarios is summarized in Table 3. This framework provides a decision-oriented overview of when each modality should be applied, based on disease stage, clinical suspicion, and specific diagnostic targets. In particular, reduced GLS may represent an early trigger for CMR, whereas elevated native T1 or ECV provides confirmation of diffuse myocardial fibrosis, and reduced CFR reflects microvascular involvement.

## 10. Future Directions: AI, Automation, Radiomics, and Trial Endpoints

Rapid advances in cardiovascular imaging technologies are expected to significantly transform the evaluation of myocardial involvement in SSc. Emerging developments in artificial intelligence (AI), automated image analysis, and quantitative imaging biomarkers offer the potential to improve the detection, characterization, and prognostic assessment of SSc cardiomyopathy. These innovations may facilitate more precise identification of subclinical myocardial disease and support the development of imaging-based endpoints for clinical trials [18,27]. One of the most promising developments is the integration of AI into cardiovascular imaging workflows. AI-based algorithms can automate image segmentation, quantify cardiac chamber volumes, and analyze myocardial deformation parameters with high reproducibility. Such automated approaches may reduce observer variability and enable large-scale analysis of imaging datasets [43,60]. In diseases such as SSc, where myocardial abnormalities may be subtle and heterogeneous, AI-assisted analysis could enhance the sensitivity of imaging techniques for detecting early myocardial involvement [18,28]. Automation is particularly relevant for techniques such as speckle-tracking echocardiography and CMR parametric mapping, where quantitative measurements of myocardial deformation or tissue characteristics are increasingly used as imaging biomarkers. Standardization of image acquisition and post-processing has already been emphasized by international imaging societies to improve the reproducibility and clinical applicability of advanced imaging techniques [21,43]. In echocardiography, consensus documents on myocardial mechanics and deformation imaging highlight the importance of standardized methodologies for improving clinical reproducibility and cross-vendor comparability [32,33,51]. Radiomics represents another emerging field that may significantly expand the diagnostic and prognostic capabilities of cardiovascular imaging. Radiomics involves the extraction of large numbers of quantitative imaging features that characterize tissue heterogeneity, texture, and spatial patterns. These features can then be analyzed using machine learning techniques to identify imaging signatures associated with specific pathological processes or clinical outcomes. In the context of SSc, advanced quantitative imaging approaches may reveal subtle myocardial abnormalities that are not readily visible with conventional visual analysis [34,69,110]. In addition to improving diagnostic capabilities, advanced imaging techniques are increasingly being considered as potential surrogate endpoints in clinical trials of SSc. Because myocardial involvement in SSc often progresses slowly and may remain clinically silent for long periods, imaging biomarkers may provide sensitive markers of disease progression or therapeutic response. Quantitative parameters such as myocardial strain, native T1 values, ECV fraction, and myocardial perfusion measurements may therefore serve as objective endpoints in studies evaluating antifibrotic or immunomodulatory therapies [19,73,111]. Imaging-based outcome measures have also been discussed in the context of SSc therapeutic trials and disease activity assessment [41,112]. Furthermore, molecular imaging approaches may enable direct visualization of biological processes involved in myocardial remodeling. Nuclear cardiology techniques provide the ability to evaluate myocardial perfusion, inflammation, and metabolic activity, offering insights into the pathophysiological mechanisms underlying cardiomyopathy [99,113]. These approaches may provide novel biomarkers for assessing disease activity and evaluating treatment response in SSc and other inflammatory cardiomyopathies [90,91].

Overall, the integration of AI automated image analysis, radiomics, and molecular imaging into cardiovascular imaging frameworks is expected to enhance the precision and clinical relevance of imaging in SSc. These innovations may ultimately facilitate earlier detection of myocardial involvement, improve risk stratification, and support the development of imaging-guided therapeutic strategies in this complex multisystem disease [18,27,28].

From a clinical perspective, it is important to distinguish between established imaging techniques and emerging artificial intelligence–based approaches. While modalities such as echocardiography and CMR are routinely used in clinical practice, most AI-driven tools and radiomics applications remain investigational and are not yet integrated into standard diagnostic workflows. Several limitations currently restrict the widespread clinical implementation of artificial intelligence in cardiovascular imaging. These include a lack of standardization across imaging protocols and analysis methods, limited external validation of proposed models, and restricted availability of advanced computational tools in routine clinical settings. In addition, variability in data quality and the need for large, well-annotated datasets remain significant challenges. Therefore, further research and validation are required before AI-based approaches can be fully integrated into clinical decision-making in SSc. Despite these limitations, AI-based approaches hold significant potential to enhance early detection and risk stratification in the future.

## 11. Practical Imaging Protocol Proposed for Systemic Sclerosis Clinics

**Figure 4:**
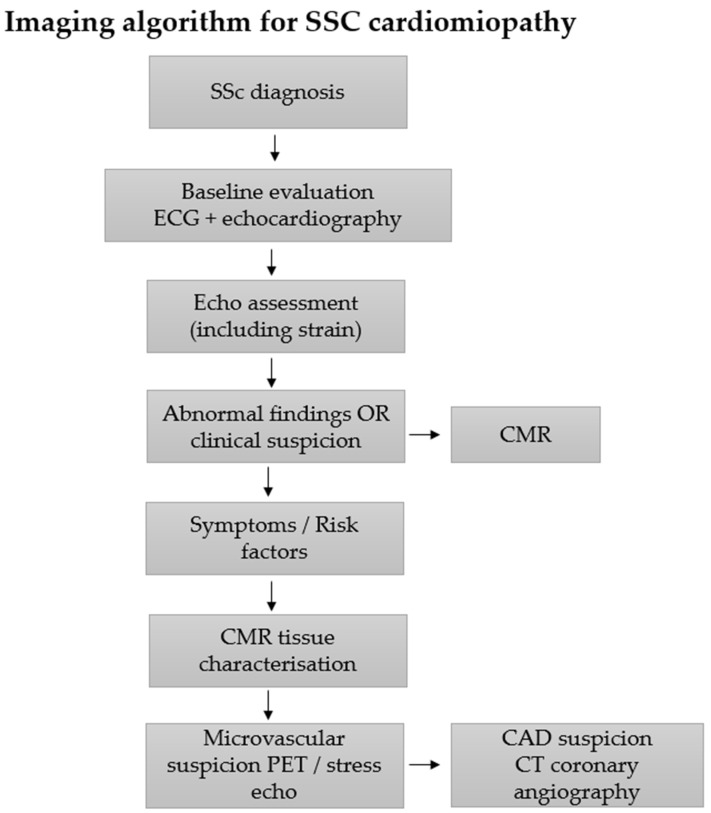
Proposed multimodality imaging algorithm for detection and evaluation of myocardial involvement in SSc.

Given the high prevalence of subclinical myocardial involvement in SSc, a structured imaging protocol is essential for early detection, monitoring of disease progression, and risk stratification. Because cardiac manifestations of SSc arise from a complex interplay of microvascular dysfunction, inflammation, and myocardial fibrosis, a multimodality imaging strategy is generally recommended to comprehensively evaluate myocardial structure, function, and tissue characteristics [18,27]. Such an approach enables clinicians to identify different components of SSc cardiomyopathy and tailor diagnostic and monitoring strategies according to individual patient risk profiles. A proposed multimodality imaging algorithm for the evaluation of myocardial involvement in SSc is illustrated in Figure 4. A stepwise approach to imaging is recommended, starting with echocardiography and progressing to advanced modalities based on clinical and imaging findings.

### 11.1. Baseline Screening

At the time of diagnosis, patients with SSc should undergo a comprehensive cardiovascular evaluation, including clinical assessment, electrocardiography, and TTE. Echocardiography represents the first-line imaging modality for cardiac screening because it allows for noninvasive assessment of ventricular size and function, diastolic parameters, pulmonary pressures, and right ventricular performance [4,35]. Standard echocardiographic measurements provide important baseline information regarding cardiac structure and function and facilitate longitudinal monitoring of disease progression.

Advanced echocardiographic techniques, particularly speckle-tracking strain imaging, may further improve the detection of early myocardial dysfunction. Reduced GLS has been shown to identify subclinical myocardial involvement in patients with SSc even when left ventricular ejection fraction remains preserved [23,24]. Because early myocardial abnormalities may precede clinically apparent cardiac disease, incorporation of myocardial strain analysis into routine echocardiographic assessment may enhance screening strategies in SSc clinics. In addition to cardiac evaluation, echocardiography also plays a key role in screening for pulmonary hypertension, a major complication of SSc. Assessment of TRV, right ventricular size, and right ventricular systolic function allows for identification of patients who may require further evaluation for PAH using right heart catheterization [25,26]. If abnormalities are detected or clinical suspicion persists, further evaluation with advanced imaging modalities, particularly CMR, is recommended.

### 11.2. Advanced Imaging Assessment

When echocardiographic abnormalities are detected or when clinical suspicion of myocardial involvement persists despite normal findings, CMR should be performed as the next-line imaging modality [18,19]. CMR is particularly indicated for the detection of myocardial fibrosis and inflammation using LGE and parametric mapping techniques [19,21]. In patients with symptoms suggestive of ischemia or in those with cardiovascular risk factors, cardiac CT may be considered to exclude obstructive coronary artery disease [78]. Nuclear imaging techniques such as PET or SPECT may be used in selected cases to assess coronary microvascular dysfunction or myocardial inflammation, particularly when other imaging findings are inconclusive [10,29]. In clinical practice, abnormal global longitudinal strain or regional strain abnormalities on echocardiography should prompt further evaluation with CMR, even in the presence of preserved ejection fraction. Nuclear imaging techniques, including PET, should be considered in selected patients with suspected coronary microvascular dysfunction or when findings from echocardiography and CMR are inconclusive. In clinical practice, a stepwise decision-oriented approach may be applied. If global longitudinal strain is reduced or regional strain abnormalities are present despite preserved left ventricular ejection fraction, further evaluation with CMR should be considered to assess myocardial fibrosis and inflammation. If CMR findings are inconclusive or if there is a high suspicion of microvascular dysfunction, PET imaging may be performed to quantify coronary flow reserve. In patients with symptoms suggestive of ischemia or relevant cardiovascular risk factors, cardiac CT should be considered to exclude obstructive coronary artery disease. Conversely, if initial echocardiographic findings are normal and clinical suspicion is low, routine follow-up with periodic echocardiography may be sufficient.

### 11.3. Longitudinal Monitoring

Because myocardial involvement in SSc may evolve gradually over time, periodic imaging surveillance is recommended. Echocardiography remains the primary modality for routine follow-up due to its availability and ability to assess ventricular function, myocardial mechanics, and pulmonary pressures [18]. Serial evaluation of parameters such as ventricular function, myocardial strain, and right ventricular performance may help identify disease progression or the development of cardiopulmonary complications. Repeat CMR may be considered in patients with previously detected myocardial abnormalities or in those with worsening symptoms or functional decline. Quantitative parameters such as native T1 values and ECV fraction may serve as useful markers for monitoring diffuse myocardial fibrosis and assessing disease progression [72,73].

### 11.4. Integrated Multimodality Strategy

In clinical practice, the optimal imaging strategy for SSc involves integration of different imaging modalities according to the clinical scenario. Echocardiography is generally used for initial screening and routine monitoring, while CMR provides detailed tissue characterization and confirmation of myocardial involvement. Nuclear imaging and cardiac CT may be employed in selected patients to evaluate microvascular ischemia, inflammatory activity, or CAD [18,27]. Such a structured multimodality imaging protocol may facilitate earlier detection of myocardial disease, improve risk stratification, and support individualized management strategies for patients with SSc. As imaging technologies continue to evolve, the integration of advanced quantitative imaging techniques may further enhance the diagnostic and prognostic value of cardiovascular imaging in specialized SSc clinics.

## 12. Conclusions

Myocardial involvement represents an important yet frequently underrecognized manifestation of SSc. The complex interplay of coronary microvascular dysfunction, immune-mediated inflammation, and progressive myocardial fibrosis contributes to the development of SSc cardiomyopathy and may ultimately lead to ventricular dysfunction, arrhythmias, heart failure, and increased risk of sudden cardiac death [1,5]. Importantly, myocardial abnormalities may develop long before clinical symptoms become apparent, underscoring the need for sensitive diagnostic approaches capable of identifying subclinical disease. Advances in cardiovascular imaging have substantially improved the detection and characterization of myocardial involvement in SSc [114]. Echocardiography remains the cornerstone of initial cardiac evaluation and longitudinal monitoring, enabling assessment of ventricular function, myocardial mechanics, and pulmonary pressures [4,35]. Techniques such as speckle-tracking strain imaging have further enhanced the ability to detect subtle impairments in myocardial function that may precede overt systolic dysfunction [23]. CMR has emerged as the reference imaging modality for comprehensive myocardial tissue characterization. Techniques including LGE and parametric mapping allow for noninvasive detection of myocardial fibrosis and inflammation, providing valuable insights into the underlying pathophysiological processes of SSc cardiomyopathy [19,21]. In addition, nuclear imaging techniques such as PET and SPECT can assess coronary microvascular dysfunction, inflammatory activity, and emerging molecular targets related to fibrotic remodeling [29,90]. Cardiac CT may serve as a complementary modality for excluding obstructive CAD and evaluating thoracic structural abnormalities [78]. The integration of these imaging modalities into structured multimodality algorithms enables more accurate detection of subclinical myocardial disease, facilitates monitoring of disease progression and therapeutic response, and improves risk stratification for adverse cardiovascular outcomes [18,27]. Imaging biomarkers such as myocardial strain abnormalities, diffuse myocardial fibrosis detected by CMR, and impaired CFR have demonstrated potential prognostic value and may help identify patients at increased risk of cardiac complications [16,23]. Future developments in cardiovascular imaging, including AI–assisted analysis, radiomics, and novel molecular imaging tracers, may further enhance the ability to detect early myocardial involvement and quantify disease activity. These advances may also facilitate the use of imaging biomarkers as surrogate endpoints in clinical trials evaluating emerging therapies for SSc [73,105]. In conclusion, myocardial involvement in SSc represents a multifaceted and clinically significant complication that requires a comprehensive diagnostic approach. Multimodality cardiovascular imaging plays a central role in the detection, characterization, and monitoring of cardiac disease in SSc and is increasingly recognized as an essential component of contemporary patient management. Continued research integrating advanced imaging techniques with clinical and molecular data will be crucial for improving early diagnosis, refining risk stratification, and ultimately enhancing outcomes for patients with SSc.

## Figures and Tables

**Table 1:** Imaging modalities in SSc cardiomyopathy.

Modality	Main Targets	Strengths	Limitations
Echocardiography	Ventricular function, myocardial strain, pulmonary hypertension	Widely available, non-invasive	Limited tissue characterization
CMR	Myocardial fibrosis, inflammation, ventricular function	Gold standard for myocardial tissue characterization	Cost, availability
PET	Inflammation, myocardial perfusion	Molecular imaging capability	Limited availability
CT	Coronary anatomy, extracardiac structures	Reliable exclusion of CAD	Radiation exposure

Abbreviations: CMR, cardiovascular magnetic resonance; PET, positron emission tomography; CT, computed tomography; CAD, coronary artery disease.

**Table 2:** Imaging biomarkers associated with myocardial involvement and adverse cardiovascular outcomes in systemic sclerosis.

Biomarker	Modality	Clinical Significance
Reduced GLS	Echocardiography	Early myocardial dysfunction and increased cardiovascular risk
LGE	CMR	Myocardial fibrosis associated with arrhythmia risk
Elevated native T1	CMR	Diffuse myocardial fibrosis
Increased ECV	CMR	Disease severity and myocardial remodeling
Reduced CFR	PET	Coronary microvascular dysfunction

Abbreviations: GLS, global longitudinal strain; LGE, late gadolinium enhancement; CMR, cardiovascular magnetic resonance; ECV, extracellular volume; CFR, coronary flow reserve.

**Table 3:** Clinical positioning of multimodality imaging in systemic sclerosis cardiomyopathy.

Clinical Scenario	Preferred Modality	Key Parameter	Clinical Role
Screening/early detection (asymptomatic patients)	Echocardiography (STE)	GLS	Detection of subclinical myocardial dysfunction
Suspected myocardial involvement despite normal LVEF	Echocardiography → CMR	Reduced GLS → T1/ECV	Trigger for advanced tissue characterization
Tissue characterization/fibrosis assessment	CMR	LGE, native T1, ECV	Identification of focal and diffuse fibrosis
Suspected inflammation/active disease	CMR ± PET	T2 mapping, FDG uptake	Detection of myocardial inflammation
Suspected microvascular dysfunction	PET (or stress echo)	CFR	Quantification of coronary microvascular impairment
Suspected ischemia/CAD exclusion	Cardiac CT	Coronary anatomy	Exclusion of obstructive CAD
Risk stratification (arrhythmias, HF)	CMR ± echo	LGE, GLS	Prognostic assessment
Inconclusive or discordant findings	Multimodality approach	Integrated parameters	Comprehensive evaluation

Abbreviations: STE, speckle-tracking echocardiography; GLS, global longitudinal strain; CMR, cardiovascular magnetic resonance; LGE, late gadolinium enhancement; ECV, extracellular volume; PET, positron emission tomography; CFR, coronary flow reserve; CT, computed tomography; CAD, coronary artery disease; HF, heart failure.

## Data Availability

No new data were created or analyzed in this study. Data sharing is not applicable to this article.

## Abbreviations

The following abbreviations are used in this manuscript:

AI	Artificial Intelligence
CAD	Coronary Artery Disease
CMR	Cardiovascular Magnetic Resonance
CFR	Coronary Flow Reserve
CT	Computed Tomography
ECV	Extracellular Volume
FDG	Fluorodeoxyglucose
GLS	Global Longitudinal Strain
LGE	Late Gadolinium Enhancement
PAH	Pulmonary Arterial Hypertension
PET	Positron Emission Tomography
SPECT	Single-Photon Emission Computed Tomography
SSc	Systemic Sclerosis
TAPSE	Tricuspid Annular Plane Systolic Excursion
TDI	Tissue Doppler Imaging
TRV	Tricuspid Regurgitation Velocity
TTE	Transthoracic Echocardiography
